# Treatment of Pneumonia

**Published:** 1870-04

**Authors:** 


### Treatment of Pneumonia.

The indications for treatment are (Dr. Flint *Med. Gazette*): (1) Promote absorption, for which mercury has been discarded, although it may exert some good influence. Iodine is often used for this purpose. (2) Palliate symptoms, as want of sleep, irritability, etc. It is here important to inquire as to previous habits of intemperance. (3) Support by good diet, and if necessary, stimulants. There is no danger, of over-nutrition, although there may be of giving a diet which is indigestible. Patients often have a repugnance of food, but when ingested it is digested. There is no disease which is not benefitted by the amount of food appropriated In this case, I would give from half ounce to one ounce doses of some form of spirit. The rule for its administration is, give enough to produce its effect; see the patient three or four hours afterward, and compare symptoms, especially if the mind be clearer, the pulse increased in volume, and less compressible. If there be improvement, we have then a guide to go by. Render the system tolerant of good alimentation by tonics, as iron, quinine and alcholic stimulants. As there is no destruction of tissues, recovery is almost sure to take place.

In this disease, we have a liability to the formation of heart clot. That the liquid conditions of the fibrin in the blood is caused by the presence of ammonia, has been broached by Dr. Richardson. He has now given up the position formerly taken by him on this point. I am disposed to think his former position the right one. I have given ammonia, and, although its does not prevent heart clot in all cases, still I think there is a certain proportion of cases in which it is beneficial. I would give five grains [of the carbonate (?)] every three or four hours. Its stimulating effects would be very beneficial.—*Med. Archives.*

